# Anserine, Balenine, and Ergothioneine: Impact of Histidine-Containing Compounds on Exercise Performance—A Narrative Review

**DOI:** 10.3390/nu17050828

**Published:** 2025-02-27

**Authors:** Maciej Jędrejko, Katarzyna Kała, Bożena Muszyńska

**Affiliations:** Department of Medicinal Plant and Mushroom Biotechnology, Medical College, Jagiellonian University, 9 Medyczna Street, 30-688 Kraków, Poland; maciej.jedrejko@proton.me (M.J.); k.kala@uj.edu.pl (K.K.)

**Keywords:** anserine, balenine, beta-alanine, carnosine, ergothioneine, exercise performance, histidine, antioxidants, anti-aging

## Abstract

Histidine is an amino acid which plays a critical role in protein synthesis, muscle buffering during anaerobic exercise, and antioxidation. It also acts as a precursor to carnosine, a dipeptide that enhances physical performance by being present in fast-contracting muscle fibers and contributing to buffering capacity. Recent studies have examined other histidine-containing compounds, such as anserine, balenine, and ergothioneine, to assess their potential benefits for physical activity. This narrative review focuses on the literature about the effects of dietary supplementation with these histidine-containing compounds on exercise capacity in animals and humans. The findings indicate that anserine may improve physical performance and reduce fatigue, particularly in quick, repetitive activities. Although balenine has been less extensively studied, it has shown promise in enhancing muscle regeneration and antioxidative defense in animal models. Ergothioneine, a sulfur-containing histidine derivative, displayed antioxidant and anti-inflammatory properties in both animal and human studies, suggesting its potential role in reducing exercise-induced oxidative stress and aiding recovery. The diversity of the presented studies and their limitations do not provide an opportunity to confirm the ergogenic properties of the histidine-containing compounds studied. Nevertheless, supplementation with anserine and ergothioneine shows promise for enhancing physical performance and recovery, though further research is required to better understand their mechanisms and optimize their use in sports and exercise.

## 1. Introduction

Histidine is an amino acid which is considered essential for infants. In contrast, it is considered non-essential for adults, except for uremic patients [1,2]. Histidine plays an important role in protein synthesis, but it is not produced by the human body. The primary dietary sources of histidine are animal products with high protein content, such as beef, chicken, and fish. A particularly high content, 109 mg/kg, is found in dried flakes of Bonito tuna (Katsuwonus pelamis). Slightly lower levels of histidine can be found in vegetable protein sources, such as beans, peas, and soybeans [3,4,5]. The average daily dietary intake of histidine is about 800 mg/day, while the daily requirement is approximately 11–14 mg/kg/day. This amino acid plays an important role in the context of physical activity. Histidine functions as an intracellular buffer in muscles, especially during anaerobic exercise. Additionally, histidine has antioxidant properties, including the ability to chelate metal ions and capture oxygen and nitrogen free radicals [2,4].

**Figure 1:**
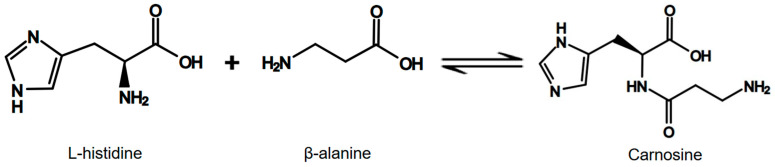
Carnosine synthesis reaction.

A particularly relevant aspect of exercise and sports is that histidine is a precursor to carnosine (β-alanyl-L-histidine). Carnosine is synthesized by combining histidine with β-alanine, with the participation of the catalytic enzyme carnosine synthase, as shown in Figure 1. Endogenous carnosine production occurs in muscles and certain areas of the brain [6].

Studies suggest that supplementation with β-alanine increases muscle carnosine content. It has a significant effect on exercise performance. This effect is caused by intercellular buffer properties, which explains the use of β-alanine by individuals and professional athletes [7,8,9].

Carnosine is a histidine-containing dipeptide, the group of which is referred to as imidazole dipeptides. The valuable role of carnosine in physical activity probably stems from two issues. First, it is localized in fast-contracting type II muscle fibers, which are involved in short but intense exercises. Second, as a histidine-containing dipeptide, it is likely to exhibit properties similar to those of amino acids, including buffering hydrogen ions (H^+^), chelating metal ions, exhibiting antioxidant effects, and increasing calcium sensitivity [6,10].

Carnosinases are characteristic enzymes that degrade carnosine into histidine and β-alanine. The high activity of carnosinase in human plasma significantly limits the effectiveness of carnosine supplementation [11]. Carnosinase 1 (CN1) is found exclusively in the serum in primates and the Syrian golden hamster. CN1 is expressed in the brain, liver, serum and glomerulus, while carnosine hydrolysis occurs at a pH of 8.5. Carnosinase 2 (CN2), referred to as a cytosolic non-specific dipeptidase, is commonly found except in the serum and cerebrospinal fluid. In the case of carnosinase 2, carnosine hydrolysis is possible at a pH value of 9.5. Besides this, there are other carnosinases belonging to the dipeptidase family EC 3.4.13. An example of this is anserinase (EC 3.4.13.5), found in fish [12]. The enzymatic activity of CN1 can be altered by a variety of compounds. Competitive inhibitors that inhibit carnosinase and its ability to degrade carnosine are betastatin and carnostatine. A compound called 1,10-o-phenanthroline inhibits CN1 by chelating zinc, an essential component for enzymatic activity. It is also worth mentioning that CN1 activity is affected by certain elements. Manganese increases the stability of the enzyme and can double its activity. On the other hand, cadmium, cobalt, and calcium have an inhibitory effect [13].

**Figure 2:**
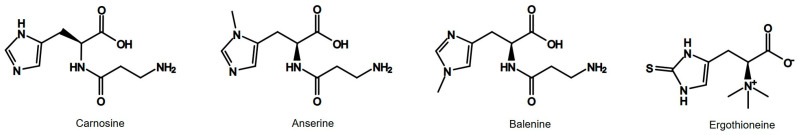
Similarities in chemical structure between carnosine and other histidine-containing compounds.

Besides carnosine, there are other histidine-containing compounds: two imidazole dipeptides—anserine and balenine—and a sulfur amino acid referred to as ergothioneine. Similarities in the chemical structure of anserine, balenine, and carnosine suggest their potential value in dietary supplementation for physically active individuals. On the other hand, the chemical structure of ergothioneine is also related to two molecules, methimazole and its prodrug carbimazole, which are registered as antithyroxine production drugs. The ergothioneine molecule shares some structural features and antioxidant activity with carnosine and anserine, which were compared in previous experiments [14,15,16,17,18]. Similarities in structure are shown in Figure 2.

It is worth mentioning that several studies support the benefits of combining anserine with carnosine in humans. Some papers indicate that acute supplementation with a dosage of 25–30 mg/kg body weight (bw) of anserine and carnosine has beneficial effects on power increases, maximal muscle contractions, ergogenic potential, and performance during short cycling sprints [19,20]. Other studies show that a 30-day supplementation protocol with a dose of 4 g of anserine and carnosine per day reduces blood lactate concentrations and the level of fatigue perceived after performing high-intensity endurance exercise [21]. The combination of anserine and carnosine is also associated with the positive effects of improving cognitive function [22]. On the other hand, in the case of resistance exercise, it may impair the body’s hormonal response to epinephrine, growth hormone, and norepinephrine [23]. Anserine is resistant to the activity of CN1, which means the enzyme does not break down anserine as quickly. As a result, the dipeptide has more time to exert a buffering effect. This is probably also the reason anserine and carnosine are used simultaneously. The degradation of carnosine by carnosinase increases the levels of L-histidine and β-alanine. The increased concentration of β-alanine, in turn, contributes to higher intracellular levels of the muscle dipeptides carnosine and anserine. Thus, this combination is believed to produce an ergogenic effect [18,24].

E. Dolan’s analysis of comparative physiology studies in various animal species seems to confirm that anserine and balenine, like carnosine, contribute to the maintenance of optimal intramuscular pH. E. Dolan also notes that the effectiveness of the buffering properties is related to the individual protein kinase A (pKa) value of each imidazole dipeptide. The pKA values for anserine and balenine are 7.04 and 7.03, respectively, and for carnosine it is 6.83. Presumably, these different pKa values mean that anserine and balenine will prove more effective at buffering the early stages of acidosis, such as at the beginning of a training session, when the pH value has begun to drop. Carnosine, on the other hand, may be a better buffer for later stages of physical activity, when the pH value is lower [25]. The similarities between anserine, balenine, and ergothioneine are not only present in terms of chemical structure. It is noteworthy that all of the mentioned histidine-containing components exhibit antioxidant activity. As for anserine, its effects are thought to relate to changes in the activity of cellular markers of oxidative stress like superoxide dismutase (SOD), catalase (CAT), glutathione (GSH), and glutathione peroxidase (GSSG) [26].

**Figure 3:**
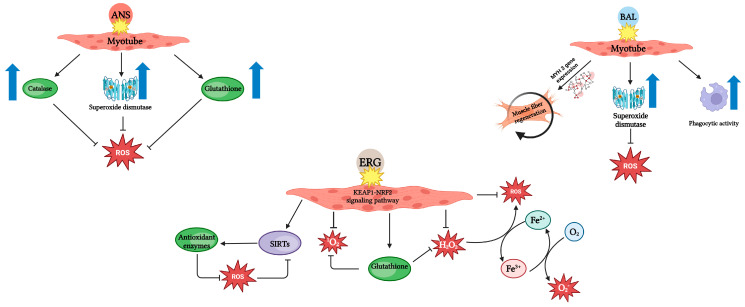
Antioxidant properties of anserine, balenine, and ergothioneine (based on [26,27,28,30,31,32,33]). Created in BioRender. Jędrejko, K. (2025) https://BioRender.com/x77d620 (accessed on 23 February 2025).

Balenine showed similar antioxidant properties. In in vitro studies, balenine led to an increase in SOD activity in C2C12 myotubes. SOD activation appeared to be significantly greater in this case than after carnosine administration. At the same time, the compound did not cause changes in CAT and glutathione peroxidase (GPx) activities [27]. Among mice given a diet rich in balenine from opah meat, increased SOD activity was observed in the skeletal muscle. In addition, balenine influenced the regulation of mitochondrial biogenesis and metabolism. Balenine included in the diet of mice was found to be important for mitigating cardiotoxin (CTX) muscle damage. Mice fed balenine showed an increase in MYH3 gene expression, which is characteristic of the muscle fiber regeneration process. Balenine increased phagocytic activity and promoted the penetration of immune cells into CTX-damaged muscles [28]. In addition, ophidine showed significantly greater efficacy in DNA protection than anserine and carnosine [29]. The antioxidant properties are shown in Figure 3.

Ergothioneine is being mentioned more frequently in the context of anti-aging and healthy aging. Bruce N. Ames has referred to ergothioneine as the “longevity vitamin”, a deficiency of which may prove important in accelerating the aging process and the development of many chronic diseases associated with old age [30].

The anti-aging effects of ergothioneine relate to its antioxidant potential. On the one hand, ergothioneine participates in the promotion of S-Adenosylmethionine (SAM) synthetase enzyme activity, which increases the catalytic activity of epigenetic methyltransferases, e.g., DMT, HMT, METTL. The increase in SAM synthetase activity is made possible by high levels of cellular GSH redox, which occur due to ergothioneine’s interaction with the KEAP1-NRF2 signaling pathway [31].

Animal studies indicate that ergothioneine can dose-dependently regulate the pathways of further antioxidant genes, e.g., heme oxygenase-1 (HO-1) or SOD [32].

On the other hand, ergothioneine, by uptaking reactive oxygen species (ROS), prevents auto-oxidation reactions of free cellular copper and iron ions. Oxidation leads to the formation of hydroxyl radicals, which can cause biomolecular damage. Thus, ergothioneine prevents the activity of demethylases that cause epigenetic demethylation from increasing [31].

The anti-aging effect of ergothioneine was observed in an in vitro study, in which it delayed the aging process of endothelial cells as a result of high glucose levels. This was a result of ergothioneine’s effects on, among other things, the levels of expression of sirtuins (SIRTs) belonging to the histone deacetylase family [32].

SIRT6 is particularly important in the context of longevity, as they regulate oxidative and inflammatory processes, facilitate DNA repair, and promote genomic stability. Regulation of SIRT-6 expression is related to the previously mentioned KEAP1-NRF2 signaling pathway [31,33]. Thus, it can be presumed that ergothioneine, as an antioxidant, may also affect epigenetic modifications of histones, DNA, and RNA. This is a valuable research direction for the future, in terms of physical activity and the “longevity” of athletes.

The aim of this work was to study the effects of dietary supplementation with other histidine-containing compounds on exercise performance in animal and human studies.

## 2. Research Selection Criteria

This narrative review was conducted between December 2023 and June 2024 and used Pubmed. To evaluate the available literature, we employed the following search terms: “anserine”, “balenine”, “ergothioneine”, “ophidine”, “exercise”, “performance”, “sport”, “athlete”, “workout”, “training”, “capacity”, “regeneration”, “antioxidation”, “anti-aging”, “regeneration”, “oxidative stress”, “intercellular buffering”, “strength”, “endurance”, “muscles”. Keywords were combined with Boolean operators (“AND” and “OR”). The initial search yielded 805 results (*n* = 805).

In the PubMed database, we additionally filtered the search according to two criteria. The first was “Text Availabilty”, including only “Full” or “Full Text” publications. The second was “Article Type”, including “Bibliography”, “Case Reports”, “Classical Article”, “Clinical Conference”, “Clinical Study”, “Clinical Trial”, “Clinical Trial, Veterinary”, “Collected Clinical Trial”, “Evaluation Study”, “Government Publication”, “Meta-Analysis”, “Randomized Controlled Trial”, “Randomized Controlled Trial Veterinary”, “Research Support, Non-U.S. Gov’t”, “Research Support, U.S. Gov’t, Non-P.H.S.”, “Research Support, U.S. Gov’t, P.H.S.”, “Research Support, U.S. Gov’t”, “Review”, “Systematic Review”. Along with the filters, there was an evaluation of the results based on the titles, abstract information, and keywords used in the publication. For manual evaluation, 430 publications (*n* = 430) were included, among which only 4 (*n* = 4) complied with the criteria for inclusion in the review. The secondary search of PubMed and an additional source, Google Scholar, provided eight supplementary records (*n* = 8). The final review included 12 publications (*n* = 12).

Research manuscripts reporting large datasets that are deposited in a publicly available database should specify where the data have been deposited and provide the relevant accession numbers. If the accession numbers have not yet been obtained at the time of submission, they should state that they will be provided during review. They must be provided prior to publication.

Interventionary studies involving humans or animals, and other studies that require ethical approval, must list the authority that provided approval and the corresponding ethical approval code.

Following the searches on PubMed and Google Scholar, a total of 12 records were identified. Among them, five studies were related to anserine supplementation, one to balenine, and six to ergothioneine. In addition, 7 of the 12 studies involved human subjects. Details about the collected studies are summarized in Table 1.

## 3. Impact of Anserine on Exercise Performance

Anserine (β-alanyl-N-π-methyl-L-histidine) is the main histidine dipeptide found in the skeletal muscle tissue of various animals, including salmon, lions, kangaroos, tuna, and trout. In the daily diet, anserine is found in fish, beef, and chicken [24,46]. Figure 2 shows the chemical structure of anserine.

The skipjack tuna, commonly known as bonito fish, is one of the main sources of anserine. Moreover, bonito muscles serve as the raw material for producing fish extracts rich in anserine (SEAns). Notably, the membrane separation process applied to bonito meat increases the concentration of anserine from 1.38% to 10.6%. Further purification processes can achieve anserine hydrochloride with a purity of 98% [47]. SEAns has been used in several studies evaluating the effects of anserine on physical activity. The doses referenced in these research papers are derived from a study evaluating the effects of SEAns on the eye and ocular fatigue, where participants were administered 400 and 1300 mg of SEAns, equivalent to 120 and 390 mg of anserine, respectively [48].

The creatine phosphokinase (CPK) concentration value is an indicator of the level of muscle damage after physical activity. A study involving 26 professional soccer players found that high CPK levels go hand in hand with athletes’ complaints of persistent muscular fatigue after matches [49]. Physical activity contributes to increased levels of cortisol, a stress hormone, which can prove detrimental in terms of controlling and regulating energy metabolism and exercise performance [50]. Elevated cortisol levels are positively correlated with poorer performance in golfers [51] and impaired serve performance in tennis players [52]. Moreover, hypercortisolemia, as a result of chronic overtraining or exhaustive exercise, can have a negative impact on the physiology of athletes [53]. As an example, high cortisol levels contribute to increased muscle catabolism and gluconeogenesis, leading to skeletal muscle degradation. The increased rate of catabolic reactions relative to anabolic processes results in a decrease in lean muscle mass [54].

The effect of anserine on activity-related fatigue was tested in a group of 10 young volunteers. Participants were given 400 mg of anserine, equivalent to 2 g of SEAns extract. The volunteers were then instructed to perform three sets of four physical exercises: push-ups, sit-ups, back muscle strength exercises, and air-chair exercises. Endurance was measured by the time to exhaustion (TTE). The anserine intervention resulted in an increase in the TTE, allowing for a longer duration of the exercise test. In addition, participants had their blood drawn, and samples taken 60 min after the workout were evaluated for cortisol and creatine phosphokinase (CPK) levels. The concentrations of both parameters were lower in those who received anserine [34].

Visual–oculomotor skills are crucial in some sports. Baseball, basketball, tennis, and volleyball require players to monitor and intercept objects moving at high speeds. Kinetic visual acuity (KVA) determines a practitioner’s ability to identify an approaching object, enabling them to make an appropriate response, such as bouncing the ball to counterattack [55,56,57]. A group of 20 male table tennis players received 8 weeks of anserine supplementation. Participants took a daily dose of 120 mg of anserine, equivalent to 400 mg of SEAns. Their performance was evaluated by analyzing their ball strike accuracy. For this purpose, the table tennis table was divided into four zones, with each zone scored based on the difficulty of the knockback and return of serve. Long-term supplementation with anserine improved accuracy and precision in bounds, which is likely related to the improvement of KVA [35].

A dose of 120 mg of anserine has also been tested in a 7-day supplementation. The participants were nine male students experienced in middle-distance running. After a week of supplementation, the participants underwent a time test (TT) consisting of three 1500 m runs. A 15 min break followed each run before the next trial. Those taking anserine completed the runs in less time than the runners in the placebo group. In addition, anserine supplementation reduced CPK activity, white blood cell (WBC) count, and free fatty acid (FFA) levels [37].

The first animal in which traces of anserine were encountered was the goose. Anserine is a dipeptide that accumulates mainly in the skeletal muscles of birds. Chicken meat, found in forms such as broth (CB) or extract (CBX), is commonly used in studies of anserine and carnosine in the context of physical activity [21,22,23,58]. In one study, CB was administered to several subjects in a 14-member male group. CB was given 40 min before the scheduled training protocol. After an 8 min timed test (TT) of cycling, participants’ blood was analyzed. A marked increase in carnosine and anserine levels was observed in those consuming CB [21].

One recent study examined the effects of acute anserine supplementation on exercise-induced oxidative stress. A group of 10 healthy men (mean bw = 69.5 ± 2.5 kg) received anserine from CBX. Anserine was administered at doses of 15 or 30 mg/kg bw. An increase in glutathione disulfide (GSSG) levels was observed in participants taking anserine. In addition, anserine led to a decrease in glutamic–oxaloacetic transaminase (GOT) levels, with concomitant increases in glutamine–pyruvic transaminase (GPT) and creatine kinase myocardial band (CKMB) levels. Both doses of anserine had no effect on the TTE test results [38].

Muscle fatigue can be evaluated with an electromyogram (EMG). An increase in the EMG wave angle indicates a high level of muscle fatigue. Similar conclusions can be drawn from other factors, such as a decrease in the mean power or a rapid decrease in median frequency (MDF) changes [59,60,61].

An EMG study analyzing the difference in MDF values was conducted with a group of 17 healthy men. The training protocol included an isometric exercise tolerance test (ETT) of the rectus femoris muscle. The ETT consisted of performing two exercises, lasting 5 and 3 min, respectively, with a 15 min break in between. Approximately 20 min before the test, some participants (mean bw = 75.5 ± 5.0 kg) consumed chicken broth anserine at a dose of 11 mg/kg bw. The placebo group showed higher angle values after each ETT, suggesting increased muscle fatigue. This shows that anserine is effective at mitigating fatigue resulting from intense exercise [36].

## 4. Impact of Balenine on Exercise Performance

Balenine, also known as ophidine, is often referred to as a marine dipeptide due to its high content in marine animals such as whales and opah (*Lampris guttatus*) [29,62].

The only human study conducted to date involved a group of 20 volunteers (6 women and 14 men, aged 27–31, mean bw = 69.0 ± 9.2 kg). A dietary supplement with pure, synthetically manufactured balenine was administered to some participants at a dose of 10 mg of balenine per kilogram of bw. As a blinding procedure, the other volunteers received an identical amount of maltodextrin in capsules. The criterion for the selection of participants was road cycling experience for at least 2 years with a minimum of 60 km/week. Exclusion factors were smoking, a vegetarian or vegan diet, and taking creatine, carnosine, or β-alanine 3 months before and during the study. The exercise protocol included knee extensor exercises and cycling tests, which consisted of short sprints and time trials (TT) over 4 and 20 km. The study authors assessed peak and rotational power, as well as time to completion (TTC) for the rides over the mentioned distances. In addition, they measured pH values and concentrations of lactate, bicarbonate, and glucose. Acute balenine supplementation did not result in changes to the analyzed parameters and thus did not affect exercise capacity [39].

In contrast to anserine and carnosine, the number of scientific papers evaluating balenine’s properties regarding physical activity is almost negligible. One of the studies compared the bioavailability of pure anserine, balenine, and carnosine after acute oral supplementation at doses of 1, 4, 10, or 20 mg/kg. Carnosine at the maximum doses was barely measurable. Anserine levels after 4 and 10 mg/kg were about 20 times lower than those of balenine, which was obtained with identical doses. Balenine showed significantly lower susceptibility to CN1 hydrolysis reactions, as well as significantly higher bioavailability after ingestion [63]. This suggests the need for a more detailed understanding of balenine as an ergogenic agent and an alternative to anserine and carnosine.

## 5. Impact of Ergothioneine on Exercise Performance

Ergothioneine (2-thiol-L-histidine-betaine) is a water-soluble sulfur derivative of the amino acid L-histidine, with an attached betaine fragment [64]. The relatively simple chemical structure of ergothioneine is notable because it contains the imidazole-2-thione substructure and a betaine fragment, as shown in Figure 2.

The human body cannot biosynthesize ergothioneine. Recent studies have identified a specific ergothioneine transporter (ETT) and confirmed high concentrations of ergothioneine in certain tissues and cells, such as the bone marrow, spleen, liver, eyes, and erythrocytes [65,66]. The concentration of ergothioneine in the human body significantly decreases with age, especially in individuals over 60 years old, which may correlate with the development of neurodegenerative diseases [67]. The EFSA has assessed the safety of synthetic L-ergothioneine (under the trade name Ergoneine) at a concentration of 470 mg/kg bw per day for adults, excluding pregnant and breastfeeding women [68].

Mushrooms are noted for their highest ergothioneine content. The amount of this amino acid varies depending on the mushroom species and the methods and conditions of cultivation, such as soil conditions. Oyster mushrooms are particularly noteworthy, containing between 607 and 11,800 mg of ergothioneine per kilogram of dry weight (DWT). In comparison, the fruiting bodies of Cordyceps militaris provide 382–799 mg/kg DWT, while the mycelium contains about 140 mg/kg DWT [69,70]. It has been confirmed that the concentration of ergothioneine in the blood significantly increases 2 h after consuming Agaricus bisporus [71].

A relatively high level of ergothioneine is also observed in several other foods. Fermented products are especially noteworthy, including shiitake mushrooms (1278–1775 mg/kg), tempeh (201 mg/kg), rice bran (176 mg/kg), and dried asparagus (163 mg/kg) [70].

The dried powder of shiitake mushrooms (*Lentinus edodes* extract) contains approximately 1.98 mg of ergothioneine per gram. In a study with young, healthy males, oral administration of shiitake mushroom extract (700 mg, twice per day) for 10 days prior to exercise had no effect on markers of inflammation. However, it did demonstrate antioxidant activity by regulating nitric oxide levels and thiol redox capacity resynthesis [40].

The effect of ergothioneine supplementation was evaluated in three studies conducted on Arabian stallions. In the first study, 10 out of 20 stallions received an acute dosage of ergothioneine at 0.2 mg/kg bw. The horses were then ridden by experienced riders with body weights ranging from 70.5 to 75 kg. The stallions were subjected to an exercise test consisting of a maximum 2000 m race on a typical racetrack. After the activity, blood samples were collected to evaluate hematological indices. In the stallions receiving ergothioneine, increased levels of leukocytes and neutrophils were observed, indicating the anti-inflammatory effect of this amino acid. In addition, the administration of ergothioneine contributed to an increase in erythrocyte levels, likely due to the stabilization of erythrocyte membranes and protection from peroxidation induced by exercise [41].

In the second study, ergothioneine was administered to 6 out of 12 stallions. The oral dose of ergothioneine was 0.5 mg/kg bw and was given weekly for 2 months. The horses were subjected to an 1800 m race in harsh weather conditions (high ambient temperature and humidity), and the riders weighed between 70.4 and 75.1 kg bw. After the race, the animals’ body temperature was measured rectally, and blood samples were analyzed—tests were conducted 5 min and 1 h after the race. Stallions receiving ergothioneine exhibited lower body temperatures. Additionally, ergothioneine was associated with lower values of oxidative stress markers, such as creatinine and malondialdehyde. In contrast, horses not supplemented with ergothioneine showed higher levels of glutathione peroxidase, indicating potentially greater oxidative damage to their cells [42].

Heat shock protein 70 (HSP-70) is produced in cells subjected to stressful stimuli, such as oxidative reactions from exercise. HSP-70 exhibits anti-inflammatory, immunoregulatory, and protective properties, impacting healthy cells threatened by damage-inducing processes [72,73].

A recent study on Arabian stallions examined the correlation between ergothioneine supplementation and HSP concentrations. An oral dose of 0.02 mg ergothioneine/kg bw was administered to 9 out of 18 animals daily for 4 weeks. The horses were then subjected to a 30 km endurance race. After 10 km, a 15 min break was taken, during which the animals received cold water. Riders continued to ride the stallions. Blood samples were collected both before and after the exercise test. Ergothioneine supplementation resulted in increased levels of HSP-70 and elevated concentrations of antioxidant enzymes, such as peroxidase and glutathione reductase. Additionally, lower levels of malondialdehyde were observed [44].

So far, two studies have examined the properties of ergothioneine in the context of exercise in mice. In the first study, 9 out of 18 female mice were supplemented with ergothioneine at a dose of 70 mg/kg/day for a week, while the other 9, serving as a control group, received a placebo. After supplementation, the mice underwent an exercise protocol involving a TTE test on a treadmill at 70% of their maximal aerobic speed (MAS). Within 2 h after the end of the exercise, the soleus and gastrocnemius calf muscles were examined to evaluate specific parameters. The ergothioneine intervention increased the MAS, the TTE, the number of satellite cells in the soleus muscle, and the number of global protein synthesis markers. Additionally, the applied dose of ergothioneine decreased markers of oxidative stress and inflammation [43].

MPST, or 3-mercaptopyruvate sulfotransferase, is an enzyme involved in the production of pyruvate and hydrogen sulfide in the mitochondria. As a result, MPST plays a role in electron flow and mitochondrial respiration [74].

In the second study, the amount of ergothioneine in the muscle increased during training. It is hypothesized that ergothioneine may act as an acceptor of MPST, thereby affecting exercise performance. A diet rich in ergothioneine (209 ng/mg bw) was administered to 8 out of 15 mice for 10 weeks. The mice were then subjected to an 8-week training protocol. Mice on the ergothioneine-enhanced diet experienced a 19% increase in training performance and a 28% rise in running speed. In addition, the total distance traveled by ergothioneine-fed mice was 14% longer than that of the control group [45].

## 6. Studies’ Limitations

The studies from Table 1 have some limitations. First is the significant difference between the humans and animals involved in the study. Carnosinases are responsible for the degradation of dipeptides and other histidine-containing components, but CN1 is not present in the serum of most animals [12,13]. Also, species diversity may imply completely different compounds that alter the enzymatic activity of carnosine dipeptidases. Thus, it is possible that the different results in the studies conducted may be the result of a distinct enzymatic distribution.

Another issue is the difference in the ingredients used. Only one of the seven human studies analyzed used a pure manufactured component [39], while the others were food derivatives [34,35,36,37,38,40]. It is therefore likely that other components in the food used may have had a beneficial or adverse effect on the reliability of the study conducted and the results obtained. Furthermore, in the study on anserine extracted from chicken broth, the authors did not include information on the purity of the extract [38].

There are also discrepancies in the parameters analyzed in the human studies from Table 1. Both anserine and ergothioneine have been checked for antioxidant activity. Balenine has not been evaluated for potential effects on markers of oxidative stress and biomarkers of cellular damage. Moreover, all of the studies used varied exercise protocols.

The last limitation is related to the extremely wide variety of doses used in human studies from Table 1. The study evaluating balenine used a dose of less than 100 mg/day, while most of the studies with anserine used larger doses. In this matter, ergothioneine deserves special attention. The doses of ergothioneine used in the studies in Table 1 are remarkably lower than the limits set by EFSA.

## 7. Implications for Future Studies on Anserine, Balenine, and Ergothioneine

Future research directions for the study of histidine-containing compounds should be considered in two dimensions—holistically, including all of the histidine-containing compounds discussed, and individually for each component.

In the former dimension, it would be worthwhile to further explore information on the affinity of anserine, balenine, and ergothioneine with human CN1. One study used a molecular dynamics simulation to explore the mechanism and evaluate the affinity of imidazole dipeptides with human CN1. Carnosine showed the highest affinity for CN1, which is determined by the longest amino chain and an additional hydroxyl group (-OH). The worst affinity is shown by balenine, which has a poor ability to bind to human CN1 due to its methylated imidazole ring [75]. According to the results of studies confirming balenine’s greater bioavailability than anserine and carnosine [63], this compound should prove more effective in terms of antioxidant and immune effects against human CN1. In contrast, the results of a study on balenine supplementation in humans from Table 1 show the opposite. It would be important to look for answers as to whether the lack of an antioxidant effect was due to the dose used. However, there may be other reasons why balenine cannot take full advantage of the fact of its low affinity for human CN1.

**Figure 4:**
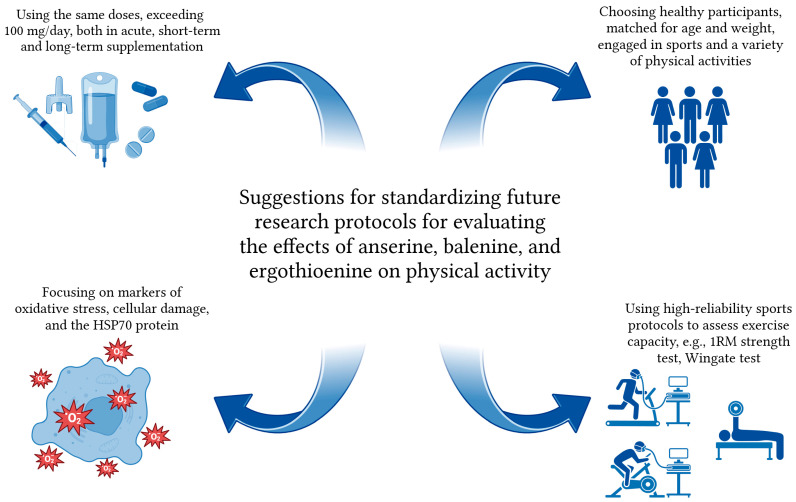
Suggestions for standardizing future research protocols. Created in BioRender. Jędrejko, K. (2025) https://BioRender.com/s52u069 (accessed on 23 February 2025).

Another important issue is to standardize the research protocol during future studies on the effects of anserine, balenine, and ergothioneine on exercise capacity. Figure 4 presents potential proposals for standardization.

In future studies, it will be important to decide whether to use pure, manufactured histidine-containing compounds or a source from the daily diet. In the second case, care should be taken to standardize, for example, the extracts used. Moreover, it would be important to use standardized daily doses, e.g., 100–200 mg. Here, we mean ad hoc, peri-workout and short- and long-term supplementation. Another issue will be the selection of suitable participants for the study. First and foremost, the focus should be on healthy individuals with similar ages, same gender, and small weight discrepancies. In addition, one criterion for selection should be the level of physical activity. To this end, it will be better to recruit participants with at least several months of training experience for the study.

A separate issue is to pay attention to the selection of oxidative stress markers and biomarkers of muscle damage under study. The evaluation of antioxidant activity should first and foremost concern balenine, for which the human study in Table 1 omitted many important markers. In addition, for future human studies, it is also worth considering the level of the HSP-70, which was one of the evaluated parameters of the study from Table 1 on ergothioneine supplementation in horses. At the same time, it should be borne in mind that, according to some scientific papers, exercise of a duration of less than 30 min is insufficient to change the activity of HSP-70 in humans [76]. Thus, the exercise protocol should consider this aspect.

In further studies, it is worth considering the use of sports protocols with proven reliability in terms of assessing exercise capacity, such as the 1RM strength test or the Wingate test [77,78]. It is also useful to have tools and tests dedicated to specific sports. For example, for football players, it could be the BEAST 90 test [79], and for rowing athletes, a training session using a rowing ergometer [80]. Future studies may consider performing tests using the inertia phenomenon and the flywheel system, which are credited with reliability and dependability when assessing the level of efficiency [81].

Another suggestion for future research is the issue of combined supplementation. This should be understood in two ways. On the one hand, it might be worth trying to evaluate anserine, balenine, and ergothioneine given simultaneously. In addition to examining their effects, perhaps it would be important to see how the individual components interact with each other. On the other hand, it would make sense to further investigate equal supplementation of anserine, balenine, and ergothioneine with carnosine, histidine, and β-alanine. In addition, it may also be worth considering combining histidine-containing compounds with other supplements that are used in sports, e.g., dietary nitrate [82], citrulline [83], creatine [84], or N-acetylcysteine [85].

Aside from the previous suggestions, it is important to check the bioavailability levels of anserine, balenine, and ergothioneine from both manufactured and natural sources. In addition to their bioavailability, it would be worthwhile to assess their antioxidant potential. Comparing the real effects on oxidative stress would perhaps provide insight into the answer of the extent to which other nutrients (e.g., those contained in mushrooms or meat) may affect the properties of histidine-containing components.

The second dimension of future research directions is closely related to specific histidine-containing components. The studies in Table 1 included anserine from two sources—fish and chicken. Supplementation with anserine from SEAns showed greater efficacy in the context of exercise capacity and physical activity parameters than CB. It is noteworthy, however, that only 1 of the 5 papers analyzed involved anserine with CB which was, in addition, administered on an acute basis. Future papers may consider testing long-term supplementation with CB-derived anserine. In addition, it may be important to evaluate different doses of anserine and compare the results from SEAns and CB supplementation in similar participants.

On the issue of balenine, there are many areas that need further exploration. Setting a minimum dose for humans with an effective effect on the antioxidant potential of cells is worth taking as a starting point. In addition to this, it seems crucial to test, in humans, balenine supplementation derived from natural sources, e.g., standardized opah or whale extract. In addition, it makes sense to validate balenine in animal studies due to the minimal number of in vivo studies.

The results from Table 1 show that there is potential in shiitake extract that is worth exploring further. For this reason, it seems justified to use higher doses of the standardized extract, thus yielding a higher content of ergothioneine. In addition, attention should be paid to other species of mushrooms, such as oyster mushrooms and cordyceps. In addition to laboratory evaluation of pure ergothioneine content, it is important to check the level of its bioavailability as well as to compare the effectiveness of these extracts with shiitake extract.

## 8. Conclusions

Carnosine is not the only histidine-containing compound that affects exercise capacity. Previous studies on human subjects indicate that anserine also shows promising results. Balenine certainly requires further research, as the current body of evidence is extremely limited. While the use of ergothioneine, a histidine-containing sulfur amino acid, has shown beneficial properties in animals, the amount of research in humans is insufficient to fully assess its effects. In addition, significant limitations in the studies included in this review make it impossible to conclude unequivocally that these histidine-containing compounds are indeed of value in supporting physical activity.

In future studies, it would be worthwhile to compare the effects of anserine, balenine, and ergothioneine supplementation on active individuals across different exercise protocols. In addition, it is important to use pure ingredients or standardized extracts in the research. This will increase the reliability of the results obtained and enable a better evaluation of their effects. The research would enable athletes to assess which imidazole dipeptides could be valuable during the preparation period for competitions. It is particularly important to focus on ergothioneine, which has recently emerged in the dietary supplement market. Assessing the ergothioneine content of popular supplements and developing a certified, safe raw material are promising research directions for the future.

## Figures and Tables

**Table 1:** Effects of anserine, balenine, and ergothioneine supplementation on exercise performance.

References	Ingredient	Participants	Dose	Duration Time	Exercise Protocols	Investigated Parameters	Results
					**In vivo human studies**		
[34]	Anserine (SEAns)	10 healthy male individuals *n* = 10	400 mg (2 g SEAns)	Acute dosage	3 × 4 physical fatigue-inducing tasks	CPK TTE Cortisol level	↓ CPK ↓ TTE ↓ Cortisol level
[35]	Anserine (SEAns)	20 male table tennis players *n* = 20	120 mg (400 mg SEAns)	8 weeks	Alternate bounce of the served ball in pairs	KVA The accuracy of the rebound of the counterattack serve	↓ KVA More points of improvement in return accuracy
[36]	Anserine (from fish)	17 healthy male individuals *n* = 17 Aged 35–40 Mean body weight 75.5 ± 5.0 kg	11 mg/kg body weight	Acute dose	Isometric ETT *m. rectus femoris*	MDF	↓ MDF
[37]	Anserine (SEAns) Placebo	9 healthy male individuals *n* = 9 Aged 19–21	120 mg (400 mg SEAns)	7 days	3 × 1500 m running TT	CPK WBCs count FFA Blood glucose Total ketone bodies Time for the 1500 m trial	↓ CPK ↓ WBCs ↓ FFA ↔ for all investigated parameters Shortening of TT in compare to baseline value, and placebo group
[38]	Anserine (chicken broth) Placebo	10 healthy male individuals *n* = 10 Aged 20–23 Mean body weight 69.5 ± 2.5 kg	15 or 30 mg/kg body weight	Acute dosage	TTE test on treadmill	Exercise performance (TTE) Oxidative stress markers (SOD, CAT, GSSG, GSH) Biomarkers of cell damage (GOT, GPT, CMKB) Hematology biomarkers of blood	↔ TTE ↔ H_2_O_2_ ↑ SOD, GSSG ↓ CAT, GSH ↑ CKMB, GPT ↓ GOT ↔ for all investigated parameters
[39]	Balenine Placebo	20 healthy individuals (6 female, 14 male) *n* = 20 Aged 27–31 Mean body weight 69.0 ± 9.2 kg	10 mg/kg body weight	Acute dosage	3 × 3 s MVC with 20 s rest 3 × 6 s maximal cycling sprints with 2 min rest 4 and 20 km TT	Peak power Peak Torque TTC 4 and 20 km pH, glucose, bicarbonate, and lactate concentration	↔ for all investigated parameters
[40]	Ergothioneine (Lentinus edodes extract/shiitake mushrooms) Placebo	14 healthy male individuals *n* = 14 Aged 21–22	1400 mg/day of L. edodes extract (2.77 mg of ergothioneine)	10 days	Exercises on treadmill: 90 min run	CK Immune cells (LC, MONO, NC, LYM) Thiol redox status Inflammatory cytokines Reactive oxygen and nitrogen species	↔ CK ↔ Immune cells ↔ GSHt ↓ GSSG ↓ IL-10 ↔ IL-1β, IL-6, TNF-α ↔ H_2_O_2_ ↑ NO (postexercise 20 min) ↓ 8-iso (postexercise 20 min and 24 h)
					**In vivo animal studies**		
[41]	Ergothioneine Placebo	20 stallions *n* = 20	0.2 mg/kg body weight	Acute dosage	Maximal race of 2000 m on a standard racetrack	Hematological parameters	↓ LC, NC, NLR ↑ EC, PCV
[42]	Ergothioneine Placebo	12 stallions *n* = 12	0.5 mg/kg body weight	2 months	Race of 1800 m on a standard racetrack	Body temperature Oxidative stress markers (AST, LDH, CREAT, SOD, CAT, GPx, MDA)	↓ Rectal temperature ↓ Oxidative stress markers
[43]	Ergothioneine Placebo	18 female mice *n* = 18	70 mg/kg bodyweight/day	7 days	TTE test on treadmill 10 min of warm-up Increase in speed run by 2 m/min every minute till 70% MAS	MAS TTE Inflammation markers (TNF-α) Markers of global protein synthesis (PI, RPS6p) Muscle protein breakdown markers Metabolic stress markers (AMPKαp) Oxidative stress markers Muscle satellite cells	↑ MAS ↑ TTE ↓ TNF-α ↑ Markers of global protein synthesis ↔ for all investigated parameters ↓ Metabolic stress markers ↔ for all investigated parameters ↑ *m. soleus* satellite cells
[44]	Ergothioneine Placebo	18 stallions *n* = 18	0.02 mg/kg body weight	4 weeks	Endurance exercise of 30 km distance Each 10 km was followed by a 15 min break and consumption of cold water	Heat Shock Protein-70 Oxidative stress markers (SOD, CAT, GPx, GR, MDA)	↑ Heat Shock Protein-70 ↑ GR, GR ↓ SOD, CAT, MDA
[45]	Ergothioneine (from diet)	15 mice *n* = 15	209 ng/mg body weight	8 weeks	VWR	Training parameters (exercise performance, running speed, total distance)	↑ for all investigated parameters

Abbreviations: ↑—increase in the value of the investigated parameter; ↓—decrease in the value of the investigated parameter; ↔—nonsignificant changes in the value of the investigated parameter; 8-iso—8-isoprostane; AMPKαp—AMPKα phosphorylation; AST—aspartate aminotransferase; CAT—catalase; CK—creatine kinase; CKMB—creatine kinase myocardial band; CPK—creatine phosphokinase; CREAT—creatinine; EC—erythrocyte count; ETT—exercise tolerance test; FFA—free fatty acids; GOT—glutamic–oxaloacetic transaminase; GPx—glutathione peroxidase; GR—glutathione reductase; GSHt—total glutathione; GSSG—oxidized glutathione; H_2_O_2_—hydrogen peroxide; LC—leukocytes; LDH—lactate dehydrogenase; LYM—lymphocytes; MAS—maximal aerobic speed; KVA—kinetic visual acuity; MDA—malondialdehyde; MDF—median frequency changes; MONO—monocytes; MVC—maximal voluntary isometric contractions; NC—neutrophils; NLR—neutrophil-to-lymphocyte ratio; NO—nitric oxide; PCV—packed cell volume; PI—puromycin incorporation; RPS6—RPS6 phosphorylation; SEAns—salmon muscle extracts rich in anserine; SOD—superoxide dismutase; TNF-α—tumor necrosis factor-alpha; TT—time trial; TTC—time to completion; TTE—time to exhaustion; VWR—voluntary wheel running; WBCs—white blood cells.
